# Genetic Diversity and Population Structure in Aromatic and Quality Rice (*Oryza sativa* L.) Landraces from North-Eastern India

**DOI:** 10.1371/journal.pone.0129607

**Published:** 2015-06-12

**Authors:** Somnath Roy, Amrita Banerjee, Bandapkuper Mawkhlieng, A. K. Misra, A. Pattanayak, G. D. Harish, S. K. Singh, S. V. Ngachan, K. C. Bansal

**Affiliations:** 1 ICAR-National Bureau of Plant Genetic Resources, Regional Station, Umiam, Meghalaya, India; 2 ICAR Research Complex for North Eastern Hill Region, Umiam, Meghalaya, India; 3 ICAR-National Bureau of Plant Genetic Resources, Pusa Campus, New Delhi, India; National Institute of Plant Genome Research (NIPGR), INDIA

## Abstract

The North-eastern (NE) India, comprising of Arunachal Pradesh, Assam, Manipur, Meghalaya, Mizoram, Nagaland, Sikkim and Tripura, possess diverse array of locally adapted non-Basmati aromatic germplasm. The germplasm collections from this region could serve as valuable resources in breeding for abiotic stress tolerance, grain yield and cooking/eating quality. To utilize such collections, however, breeders need information about the extent and distribution of genetic diversity present within collections. In this study, we report the result of population genetic analysis of 107 aromatic and quality rice accessions collected from different parts of NE India, as well as classified these accessions in the context of a set of structured global rice cultivars. A total of 322 alleles were amplified by 40 simple sequence repeat (SSR) markers with an average of 8.03 alleles per locus. Average gene diversity was 0.67. Population structure analysis revealed that NE Indian aromatic rice can be subdivided into three genetically distinct population clusters: P1, *joha* rice accessions from Assam, *tai* rices from Mizoram and those from Sikkim; P2, *chakhao* rice germplasm from Manipur; and P3, aromatic rice accessions from Nagaland. Pair-wise *F_ST_* between three groups varied from 0.223 (P1 vs P2) to 0.453 (P2 vs P3). With reference to the global classification of rice cultivars, two major groups (*Indica* and *Japonica*) were identified in NE Indian germplasm. The aromatic accessions from Assam, Manipur and Sikkim were assigned to the *Indica* group, while the accessions from Nagaland exhibited close association with *Japonica*. The *tai* accessions of Mizoram along with few *chakhao* accessions collected from the hill districts of Manipur were identified as admixed. The results highlight the importance of regional genetic studies for understanding diversification of aromatic rice in India. The data also suggest that there is scope for exploiting the genetic diversity of aromatic and quality rice germplasm of NE India for rice improvement.

## Introduction

Rice (*Oryza sativa* L.) is rich in genetic diversity at both inter- and intra-specific levels. The genetic structure of rice is well characterized (see reviews in [1,2]). In addition to the two major subspecies, *Indica* and *Japonica*, five genetically defined groups, *indica*, *aus*, *aromatic*, *temperate japonica* and *tropical japonica* have been identified with genetic markers [3,4,5,6,7]. Aromatic/ quality rice is a special class of rice with high market value due to its superior grain qualities and pleasant aroma. Aromatic accessions have been identified within *Group V* (*i*.*e*., ‘Basmati’ and ‘Sadri’ varieties), *indica* (*i*.*e*., ‘Jasmine’ varieties) and *tropical japonica* [8,9]. The centre of diversity of aromatic rice in India is the foothills of Himalayas, covering Uttar Pradesh, Bihar and the *Tarai* region of Nepal [10]. From this centre of diversity, aromatic rice germplasm dispersed to the other states of India and neighbouring countries and adapted to the local environments. The genetic structure of Indian aromatic and quality rice has been studied in the past [11,12,13]. All these studies included very few aromatic rice accessions from the North-eastern (NE) states of India and thus did not provide the actual picture of genetic diversity in aromatic and quality rice germplasm prevailing in this region.

The NE India is a large geographical area comprising of eight states namely Arunachal Pradesh, Assam, Manipur, Meghalaya, Mizoram, Nagaland, Sikkim and Tripura. This region is surrounded by Bhutan and China in the North, Myanmar in the East and Bangladesh in the Southern side. The NE India is characterized by high rainfall, humidity, varied topography, heavy natural selection pressures and environmental stresses. Being a biodiversity hotspot [14], the region is rich both in floristic and crop diversities. Rice is the principal crop of this region possessing around 72% of the total cultivated area. Rice is cultivated in upland, lowland and deep water conditions. It has been estimated that at least 10,000 indigenous cultivars are prevailing in this region [15]. The farmers of this region still grow their heirloom cultivars which not only suit to their taste but also provide crop security. The NE India is also the home to many locally adapted aromatic and quality rice land races. Despite their low-yield potential, these cultivars are grown for their high market and social values. There are prominent cultivar groups within the aromatic rice gene pool of NE India. For example, *joha*, *chakhao* and *tai* cultivars grown in the states of Assam, Manipur and Mizoram, respectively. The traditional aromatic rice cultivars of Assam are locally known as *joha*. It is a special class under *sali* or winter rice. The *joha* rice cultivars are popular for its unique aroma, good cooking qualities and excellent palatability [16]. In the state of Manipur, the aromatic rice cultivars are locally known as *chakhao* (*chak* = rice and *ahoba* or *hao* = delicious). These landraces are grown both under upland and lowland ecologies. The population genetic analysis of *chakhao* rice accessions of Manipur have been conducted recently [17]. The *tai* rice cultivars of Mizoram are aromatic in nature and are mostly cultivated under wetland conditions. The aromatic rice germplasm collected from the state of Nagaland has been phenotypically characterized. This rice germplasm has considerable variation in grain size, shape, awn and glume characteristics [18]. A considerable number of aromatic rice cultivars, namely *Krishna Bhog*, *Brimphul*, *Kalanunia* and local Basmati are grown in the states of Sikkim and Tripura. The aromatic and quality rice cultivars are mainly used for preparing special dishes in festive occasions and marriage ceremonies.

Knowledge regarding the extent of genetic variation and genetic relationships between genotypes are vital for designing effecting breeding and conservation strategies. Population genetic analysis of rice landraces collected from a region also helps in understanding the complex interaction between rice diversity and human cultivation practices and culture, as the cultivar structure is shaped by the interplay between adaptation to the local environment and artificial selection imposed by the farmers. In contrast, the global studies though provided an excellent overview of the genetic structure of cultivated rice, very often include only a few cultivars from a region and thus unable to explain the rice diversity at a local scale. A large collection of rice germplasm including the aromatic and quality rice landraces from NE India is conserved at National Genebank, National Bureau of Plant Genetic Resources (NBPGR), New Delhi. These valuable aromatic rice germplasm has not been characterized at molecular level.

In the present study, a diverse set of 107 aromatic and quality rice accessions collected from various parts of the NE India were analysed using 40 genome-wide SSR markers. The objectives were (i) to estimate the level of genetic diversity and population structure and (ii) to address the genetic classification of the NE Indian rice accession in the context of structured global rice cultivars.

## Materials and Methods

### Plant materials

**Fig 1:**
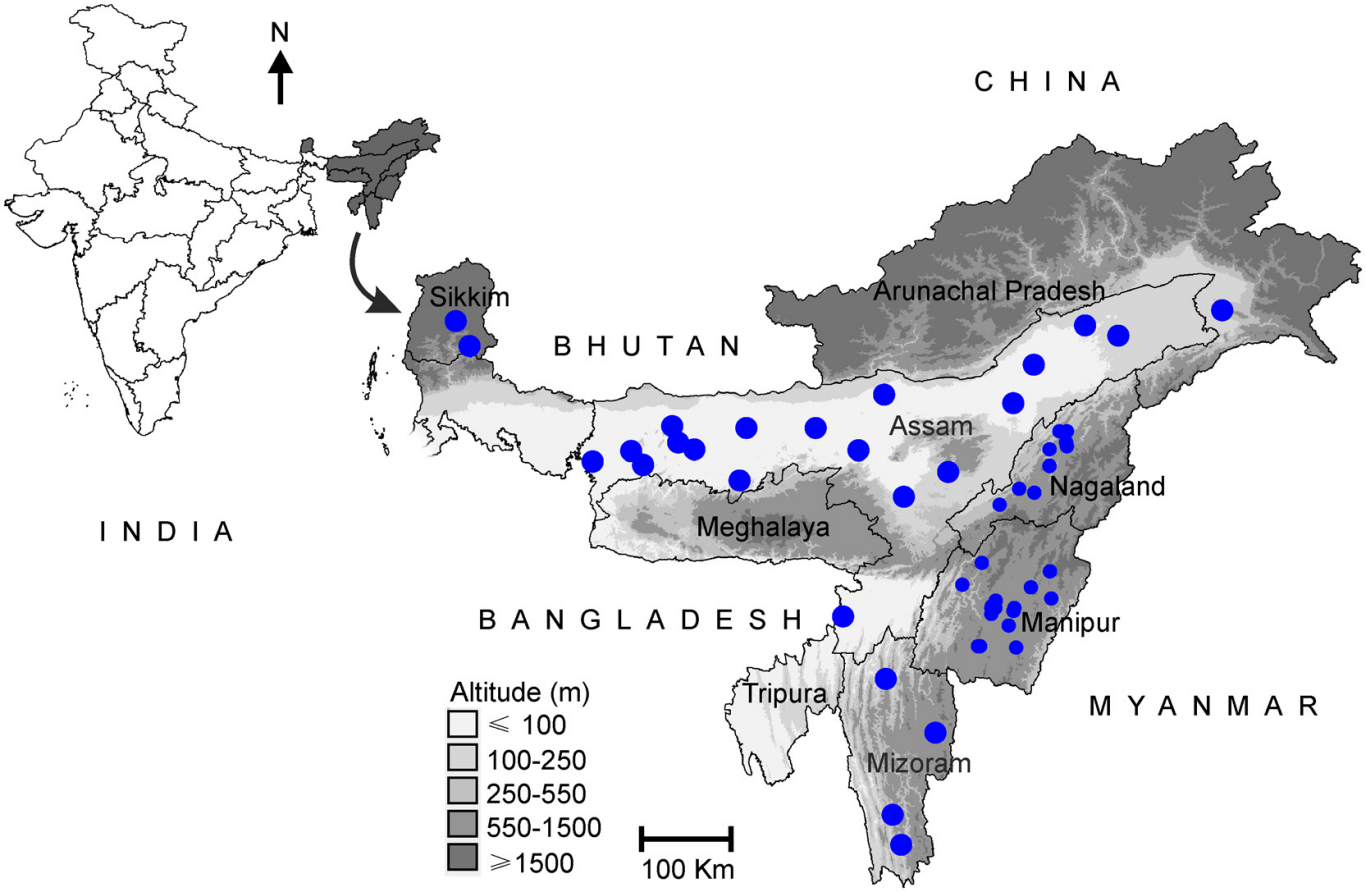
Geographical location of North-eastern region of India. The collection sites of rice landraces are indicated by circles. Smaller and bigger dots denote the collection sites with and without actual geographical coordinates, respectively.

A total of 107 aromatic/ quality rice accessions (Assam: 54, Nagaland: 21, Manipur 16, Mizoram: 9, Sikkim: 5 and Arunachal Pradesh: 2) were sampled from the National Genebank, NBPGR based on characterization data and passport information. The information on the names, accession number, collection sites, kernel length, kernel shape, aroma level and inferred group as determined with STRUCTURE [19] of each accession is presented in S1 Table. The collection sites of these rice accessions are shown in Fig 1. In addition, a set of 67 structured global rice accessions (representing *indica*, *aus*, *aromatic*, *temperate japonica*, *tropical japonica* and admixed cultivars originating from India and its neighbouring countries) were selected from the study conducted by Garris et al. [3] (S2 Table). The genotyping data for the 30 SSR markers (common with the present study) were taken from Garris et al. [3] and used for a combined analysis.

### DNA extraction and SSR genotyping

Total genomic DNA was extracted from five day-old seedlings germinated from five well developed seeds of each accession using Qiagen DNeasy plant mini kit (Qiagen, CA, USA) following the protocol provided by manufacturer. Forty SSR markers distributed across the rice genome were designed from the ‘Gramene’ marker database (http://www.gramene.org/markers/microsat/). Detailed information about these markers is given in Table 1.

**Table 1:** Summary statistics of the 40 SSR markers used in this study.

Marker	Motif	Chr	Size range (bp)	AN	Major allele		*H*_*e*_	*H*_*o*_	PIC
					Size (bp)	Frequency (%)			
RM495*	(CTG)7	1	147–159	5	159	32	0.78	0.00	0.74
RM1* ^†^	(GA)26	1	74–118	12	94	21	0.85	0.03	0.84
RM259* ^†^	(CT)17	1	148–181	16	181	20	0.88	0.01	0.87
RM5* ^†^	(GA)14	1	104–130	7	114	29	0.77	0.03	0.73
RM431* ^†^	(AG)16	1	240–252	4	252	38	0.72	0.01	0.67
RM240	(CT)21	2	109–145	13	109	45	0.74	0.01	0.71
RM213	(CT)17	2	119–153	13	137	36	0.77	0.00	0.74
RM208^†^	(CT)17	2	156–178	8	164	56	0.63	0.00	0.60
RM207	(CT)25	2	69–156	20	126	29	0.84	0.04	0.82
RM489* ^†^	(ATA)8	3	235–271	4	265	45	0.67	0.00	0.61
RM338* ^†^	(CTT)6	3	179, 182	2	179	64	0.46	0.00	0.35
RM55* ^†^	(GA)17	3	217–238	6	232	66	0.53	0.01	0.50
RM514* ^†^	(AC)12	3	237–269	8	245	50	0.68	0.01	0.64
RM124* ^†^	(TC)10	4	265–271	4	267	47	0.67	0.00	0.61
RM507* ^†^	(AAGA)7	5	254, 258	2	254	57	0.49	0.00	0.37
RM413* ^†^	(AG)11	5	69–105	8	79	31	0.75	0.01	0.71
RM178* ^†^	(GA)5(AG)8	5	115–121	4	115	85	0.27	0.00	0.26
RM334* ^†^	(CTT)20	5	143–207	12	143	18	0.87	0.00	0.86
RM26	(GA)15	5	102–114	6	106	44	0.71	0.00	0.67
RM133* ^†^	(CT)8	6	228–232	3	228	57	0.57	0.00	0.50
RM510* ^†^	(GA)15	6	109–125	5	111	30	0.75	0.01	0.70
RM190^†^	(CT)11	6	105–139	8	126	29	0.80	0.00	0.77
RM217	(CT)20	6	114–200	16	120	31	0.80	0.05	0.77
RM125* ^†^	(GCT)8	7	126–147	4	126	49	0.64	0.01	0.57
RM11* ^†^	(GA)17	7	121–143	6	121	57	0.61	0.01	0.57
RM118* ^†^	(GA)8	7	142–162	5	158	47	0.64	0.00	0.57
RM234^†^	(CT)25	7	133–164	13	133	42	0.78	0.02	0.77
RM505^†^	(CT)12	7	179–207	5	179	68	0.46	0.00	0.39
RM152* ^†^	(GGC)10	8	132–155	5	144	71	0.47	0.01	0.43
RM25* ^†^	(GA)18	8	129–147	5	129	42	0.67	0.02	0.61
RM284* ^†^	(GA)8	8	142–150	4	142	67	0.49	0.00	0.44
RM433* ^†^	(AG)13	8	219–233	8	223	74	0.45	0.00	0.43
RM223	(CT)25	8	142–165	9	152	28	0.82	0.00	0.80
RM80	(CTC)25	8	98–172	12	127	50	0.71	0.00	0.68
RM242	(CT)26	9	186–225	11	186	68	0.51	0.02	0.48
RM171* ^†^	(GATG)5	10	324–344	5	336	67	0.49	0.00	0.44
RM552* ^†^	(TAT)13	11	157–248	17	157	42	0.71	0.01	0.67
RM144* ^†^	(ATT)11	11	220–274	8	220	51	0.64	0.00	0.59
RM209	(CT)18	11	121–155	9	125	21	0.84	0.05	0.81
RM19* ^†^	(ATC)10	12	205–251	10	214	49	0.70	0.05	0.66
**Mean**		**-**	**-**	**8**	**-**	**46**	**0.67**	**0.01**	**0.62**

Notes:

*Taken from panel of 50 standard SSR markers: http://archive.gramene.org/markers/microsat/50_ssr.html.

^†^SSR markers selected for combined analysis of current genotyping data and global rice germplasm data reported in Garris et al. [3].

Chr, Rice chromosome; AN, Number of allele per locus; *H*_*e*_, Gene diversity or expected heterozygosity; *H*_*o*_, Observed heterozygosity; PIC, Polymorphism information content.

Polymerase chain reaction (PCR) was performed in a total volume of 25 μl containing 50 ng of template DNA, 0.2 mM of dNTPs, 2.5 mM MgCl2, 2.5 μl 10X PCR buffer, 2.5 pmol of each primer, 1.0 unit of *Taq* DNA polymerase and double-distilled water to make the final volume up to 25 μl. Amplification was carried out using a thermocycler (Mastercycler, Eppendorf, Hamburg, Germany) and PCR conditions were: 5 min at 94°C; 35 cycles of 30 s at 94°C 30 s at annealing temperature and 30 s at 72°C; followed by 7 min at 72°C. The amplified products were visualized by ethydium bromide stained 2.5% agarose gels in BioRad GelDoc XR system.

The molecular sizes (in nucleotide) of the amplified alleles were determined based on migration relative to the DNA ladder using the PyElph-1.4 gel image analysis software [20]. The band sizes of each marker were checked from the Gramene database (http://www.gramene.org/marker/microsat/). In case of non-amplification, we repeated the PCR to exclude technical failure and in case of failure in both PCRs a null allele was recorded. Genotype data of all accessions are given in S3 Table.

### Data analysis

The average number of alleles per locus, major allele frequency, gene diversity, heterozygosity and polymorphism information content (PIC) were calculated using PowerMarker V3.25 [21].

Population structure was examined using the Bayesian model-based approach implemented in STRUCTURE V2.3.4 [19,22]. The number of clusters (*K*) evaluated here ranged from 1 to 10. The analysis was performed using five replicate runs per *K* value, a burn-in period length of 5000, a run length of 50000, and a model allowing for admixture and correlated allele frequency. ‘Structure harvester’ programme (http://taylor0.biology.ucla.edu) was used to determine the final *K* value (*K* = 3 was optimum for this analysis) based on both the LnP(D) and Evanno’s Δ*K* [23]. Subsequently, 10 simulations at *K* = 3 were then performed with a burn-in period of 10000 and a run length of 100000. At *K* = 3, the membership coefficient from the run with the lowest likelihood value (-9442.1) was used to assign each accession to the *K* = 1 to 3 populations based on the highest membership coefficient. The STRUCTURE analysis of the combined dataset (NE Indian and global accessions) was performed following same procedures and run conditions. The graphical display of the structure results was generated using DISTRUCT programme [24].

Pair-wise values of F-statistics (*F*_*ST*_) were estimated using Arlequin V3.5 [25]. Genetic distance was calculated using the C. S. Chord distances [26], and the phylogenetic tree was constructed based on neighbour-joining (NJ) method implemented in PowerMarker. The tree was visualized using Dendroscope V3 [27]. To summarize the major patterns of variation in multi-locus data set, principal coordinate analysis (PCoA) was performed using GenAlEx V6.5 [28]. The molecular variance of sub-populations and accessions within the sub-populations were calculated using an Analysis of Molecular Variance (AMOVA) approach in Arlequin.

## Results

### Microsatellite diversity

In this study, 40 SSR markers distributed over all 12 chromosomes of rice were genotyped in 107 accessions of aromatic and quality rice landraces of NE India. The summary statistics of the 40 SSR markers are given in Table 1. All the markers showed polymorphism and a total of 322 alleles were identified. The average number of alleles per locus (AN) was 8.03, ranging from two (RM338 and RM507) to 20 (RM207). The gene diversity or expected heterozygosity (*H*_*e*_) ranged from 0.27 to 0.88, with an average of 0.67. The observed heterozygosity (*H*_*o*_) was 0.01. The polymorphism information content (PIC) for the SSR loci ranged from 0.26 (RM178) to 0.87 (RM259).

### Population structure and genetic relationships

**Fig 2:**
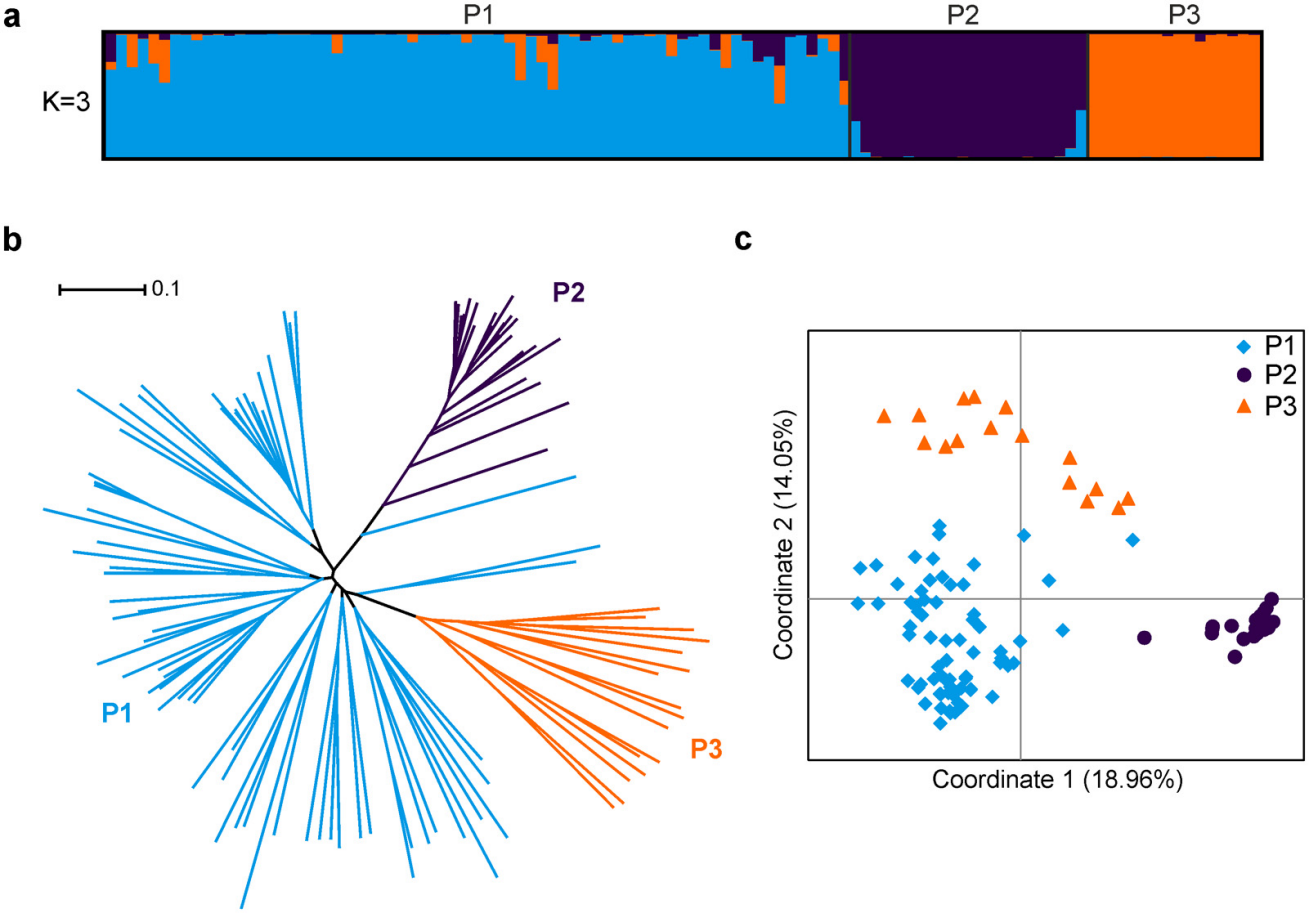
Population structure of 107 aromatic and quality rice accessions of North-eastern India. (**a**) Model-based clustering using STRUCTURE analysis. (**b**) NJ tree based on C. S. Chord genetic distance. (**c**) Principal coordinate analysis.

Bayesian analysis of population structure using the model-based approach in STRUCTURE provided support for the existence of population structure in aromatic and quality rice accessions of NE India. The LnP(D) and Evanno’s Δ*K* method supported the presence of three genetically distinct clusters (i.e., *K* = 3; S1 Fig), here denoted as P1, P2 and P3, respectively. The inferred population structure is given in Fig 2a. P1 contained 69 accessions, among which 54 were from Assam (*joha* accessions), nine were from Mizoram (*tai* accessions), five were from Sikkim, and one (cv. *Amo*) from Arunachal Pradesh. This group was dominated by accessions with short to medium kernel length, bold to medium kernel shape and high aroma. P2 had 22 accessions, most from Nagaland except one (cv. *Khaw*) from Arunachal Pradesh. They have long to extra-long kernel length, predominantly bold kernel shape and medium aroma. P3 contained 16 accessions, all from Manipur (*chakhao* accessions). This group was dominated by medium to long kernel length, medium kernel shape and medium to high aroma. The three sub-populations (P1–P3) had an *F*_*st*_ (population-specific *F*_*st*_ values calculated in STRUCTURE) of 0.208, 0.67 and 0.183, respectively with an average value of 0.354, indicating moderate population structure. Overall, 12 (11.2%) admixed accessions were found at *K* = 3.

The neighbour-joining (NJ) tree and principal coordinate analysis (PCoA) further confirmed the STRUCTURE results. The tree model-based groups (P1–P3) clearly separated in NJ tree (Fig 2b). Note that the accessions of P1 further divided into four sub-clusters. The largest genetic distance (0.66) was noted between P2 and P3, while, the genetic distances between P1 vs P2 and P1 vs P3 were similar (0.53 and 0.55, respectively). In the PCoA (Fig 2c), the first two eigenvectors clearly separated the accessions into three groups, similar to those observed in STRUCTURE analysis and NJ tree.

The analysis of molecular variance (AMOVA) among populations indicated that 30.5% of the variation was due to differences among groups with the remaining 68.2% due to difference within groups (Table 2). Around 1% of the total genetic variance was explained by differences at the individual level. Pair-wise estimates of *F*_*ST*_ (S4 Table) indicated that the highest degree of differentiation was between P2 and P3 (0.453), and the lowest was between P1 and P2 (0.223).

**Table 2:** Analysis of molecular variance of aromatic and quality rice landraces of North-eastern India.

Source	df	SS	CV	% Total	P value
Among Populations	2	571.5	4.8	30.54	< 0.0001
Among Individuals	104	2228.7	10.6	68.2	< 0.0001
Within Individuals	107	21.0	0.2	1.26	< 0.0001
Total	213	2821.1	15.6		

Notes: df, Degrees of freedom, SS, Sum of squares, CV, Variance component estimates, % Total, percentage of total variation.

### Genetic diversity

The amount and organization of genetic diversity differed among the model-based populations (Table 3). The highest mean major allele frequency was present in P2 (0.819) and the lowest was found in P3 (0.507). The AN was the highest in accessions of P1 (6.13) and the least in accessions of P2 (2.45). Genetic diversity varied slightly among P1 and P3 (*H*_*e*_ = 0.601 and 0.61, respectively; PIC = 0.56 in each population). The genetic diversity in P2 was comparatively low (*H*_*e*_ = 0.253; PIC = 0.227).

**Table 3:** Summary statistics of microsatellite diversity in 107 aromatic and quality rice landraces of North-eastern India.

Population	N	AN	MAF	*H*_*e*_	*H*_*o*_	PIC
P1	69	6.13	0.188–0.899 (0.524)	0.187–0.879 (0.601)	0.0–0.073 (0.013)	0.127–0.867 (0.56)
P2	22	2.45	0.381–1.0 (0.819)	0.0–0.678 (0.253)	0.0	0.0–0.638 (0.227)
P3	16	4.60	0.188–0.938 (0.507)	0.117–0.885 (0.61)	0.0–0.063 (0.01)	0.11–0.874 (0.56)
Total	107	8.05	0.179–0.849 (0.464)	0.271–0.883 (0.665)	0.0–0.047 (0.01)	0.259–0.873 (0.625)

Notes: N, Number of accessions; AN, Number of allele per locus; *H*_*e*_, Gene diversity or expected heterozygosity; *H*_*o*_, Observed heterozygosity; PIC, Polymorphism information content.

### Combined analysis of NE Indian and global rice accessions

To establish genetic grouping of the aromatic and quality rice landraces from NE India, a combined dataset including genotyping data from Garris et al. [3] was subjected to STRUCTURE analysis (174 genotypes, 30 SSR markers). Since a total of 169 SSR markers were originally used by Garris et al. [3] to infer the population structure, and only 30 of them were included in the combined dataset, the 67 global rice accessions were independently reanalysed to assess the impact of the reduction in marker number. No significant differences were observed in the structure outputs produced by the 30 SSR data matrices (*K* = 1–6) and the accessions were divided into same groupings described by Garris et al. [3] (data not shown). For all subsequent analyses, the global accessions were labelled following the grouping proposed by Garris et al. [3].

**Fig 3:**
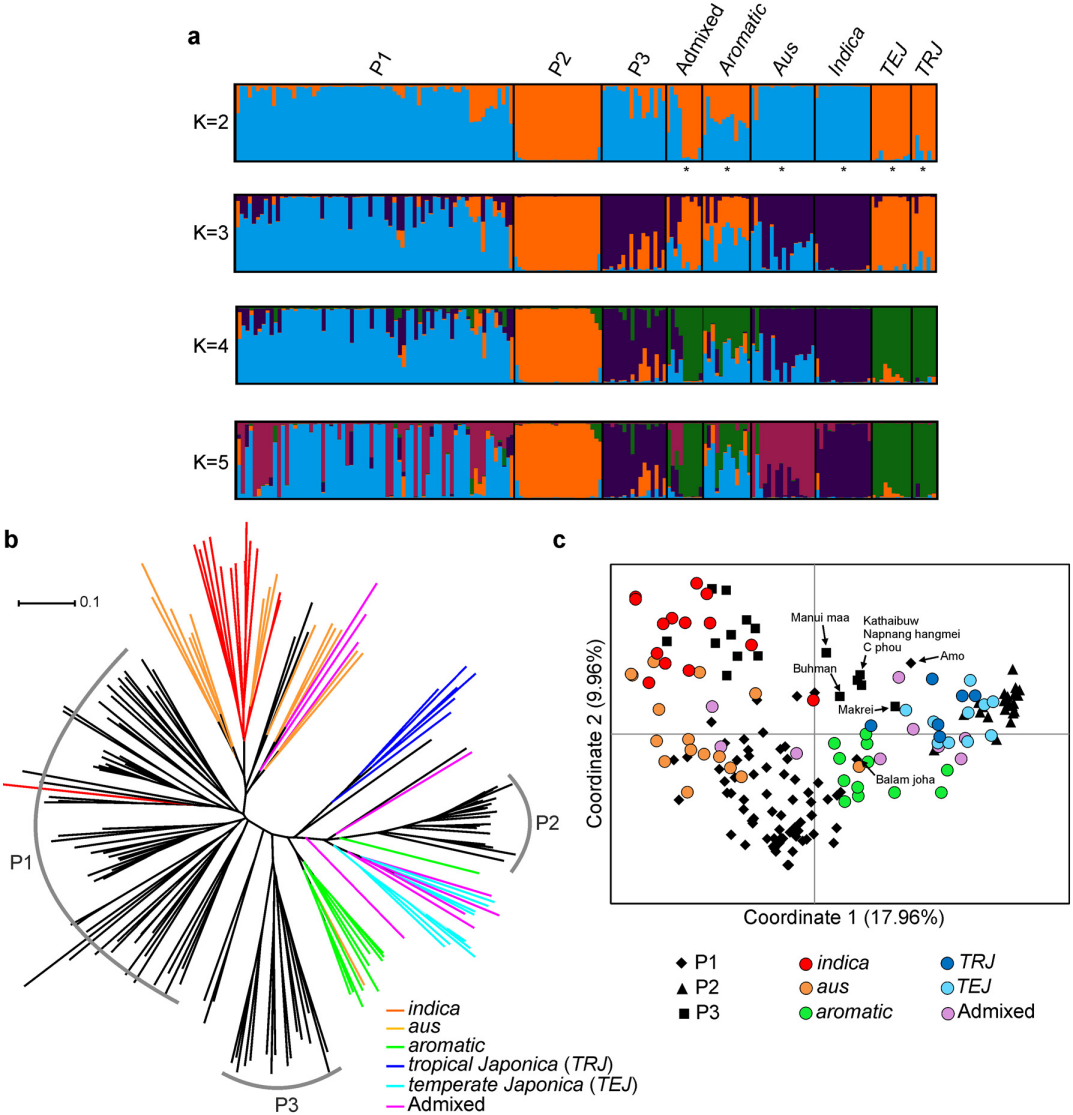
Population structure of the combined samples of North-eastern India and global rice cultivars. (**a**) Model-based population assignment using STRUCTURE analysis. (**b**) NJ tree based on C. S. Chord genetic distance. (**c**) Principal coordinate analysis.

It was observed that LnP(D) value increased with K from 1 to 10 (S2a Fig), with highest log likelihood score at *K* = 2. The procedure of Evanno et al. [23] also detected the maximal Δ*K* at *K* = 2 followed by *K* = 3. The Δ*K* value decreased with increased *K*, and no peak of Δ*K* was observed at *K* > 4 (S2b Fig). Since there was no clear indication of which *K* value (except *K* = 2) providing the best fit for the data, the population sub-groups were examined for biological relevance from *K* = 2–5 (Fig 3a). At *K* = 2, two major groups corresponding to two rice subspecies (*Indica* and *Japonica*) were apparent within the population. Out of 174 accessions, 100 (57%) were *Indica*, 42 (24%) were *Japonica* and 32 (18%) were admixed. Whereas, within NE Indian accessions 68 (63%) were *Indica*, 22 (21%) were *Japonica* and 17 (15%) were admixed (S1 Table). All the aromatic rice accessions from Nagaland were identified as *japonica*. The aromatic rice accessions from Mizoram were found to be admixed. Some of *chakhao* accessions (*Kathaibuw*, *Napnag hangmei*, *Chakhao phou*, *Makrei* and *Manui maa*) also identified as admixed. Note that these *chakhao* accessions were collected from the hill districts of Manipur. The clustering pattern obtained for *K* = 3 revealed an association between: (i) group P3 (*chakhao*) and *indica* accessions; and (ii) group P2 and *japonica* accessions. Admixed individuals seem to be prevalent in group P1 (Fig 3a). The grouping of *indica*,*aus*, *aromatic* and *japonica* accessions becomes apparent at *K* = 5. At this level, group P1 showed a closer affiliation to the *aus* and *aromatic* accessions. Most of the *chakhao* accessions of P3 showed clear association with *indica* accessions, while few identified as *indica/japonica* admixture. The aromatic accessions of Nagaland differentiated from the *japonica* cultivars at *K* = 4 and 5 (Fig 3a).

The grouping of the NE Indian aromatic rices in relation to global rice cultivars was also analysed in NJ tree and PCoA. In the NJ Tree the rice cultivars of model-based groups P1 showed a closer affiliation to the *aus* and *indica* group (Fig 3b). The accessions of P3 were clustered close to *aromatic* group. The accessions of P2 showed closer affiliation to the *temperate japonica* group and admixed accessions (Fig 3b). The results of PCoA supported the STRUCTURE results obtained at *K* = 2 (Fig 3c). Moreover, the cluster analysis of 107 NE Indian aromatic rice accessions using 30 SSR data matrices showed similar grouping to that of previous analysis using 40 SSRs (data not shown).

## Discussion

### Genetic diversity

Genetic diversity and population structure of NE Indian rice germplasm has been analyzed in past using molecular markers [29,30,31,32]. In a landmark study, Choudhury et al. [33] characterized 6,984 NE Indian rice accessions from National Genebank using 36 SNP markers. Despite the availability of tremendous diversity, very little is known about the amount and distribution of genetic diversity and population structure within aromatic and quality rice germplasm from NE India. Knowledge of the genetic structure within this rice germplasm will be of great value for utilization and development of improved cultivars. The past studies on the genetic diversity of Indian aromatic and quality rice cultivars included only a few (3–5) genotypes from NE India [11,13]. Thus these studies were unable to explain the actual diversity prevailing in this region. Recently, we have reported the population structure of aromatic (*chakhao*) rice landraces collected from Manipur [17]. Therefore, the present study is the first effort to characterize a major portion of aromatic and quality rice diversity available in NE India.

Analysis of genetic diversity parameters indicated a high level of variability in the NE Indian aromatic and quality rice collection. The mean number of alleles per locus (8.03) was similar to the estimates reported for 52 Indian aromatic rice cultivars (7.8) [13] and 83 accessions of rice cultivars from Eastern and NE Indian states (7.9) [32]. However, the average number of alleles per locus for this study was lower than the estimated recorded in the diverse sets of global rice accessions [34,35,36]. In this study the average PIC value (0.62) was similar to the values obtained for Indian aromatic and quality rice cultivars (0.6) [13] and for a subset of 26 aromatic rice cultivars from West Bengal, India (0.65) [32]. The gene diversity index in the NE Indian rice landraces (0.67) was at par with the values reported for indigenous rice varieties of NE India (0.78) [31] and 234 global rice accessions from 34 countries (0.70) [3]. Although allelic diversity and gene diversity index could be used as indicators of genetic variation, such estimates are relative and largely depend on the number of polymorphic loci and relatedness of genotypes included [37]. Despite their marginal location, aromatic and quality rice landraces from NE India exhibited high levels of genetic diversity. The diverse nature of these landraces may be a reflection of the prevalent diverse agro-climatic, ethnic and eco-geographical features of the region.

### Genetic structure

The Indian aromatic and quality rice germplasm is comprised of small-, medium-, and long-grain types with mild to strong aroma [38]. Based on conventional taxonomy, Indian aromatic rices have been classified as *indicas* [10]. Subsequent studies based on isozyme and SSR markers have shown that most of the aromatic rice cultivars of the Indian sub-continent, including Basmati types, are identifiable as a genetically distinct cluster [3,8,11,12]. Jain et al. [13] reported two major groups among a set of 52 Indian aromatic and quality rice accessions based on SSR marker analysis. They classified short-grained *joha* rice cultivars of Assam as aromatic *indicas* and proposed that these cultivars might have evolved *via* hybridization between *indica* and Basmati rice varieties. In the present study, population structure analysis revealed three subpopulations (P1–P3) out of which the majority of accession included in P1. This grouping is well in agreement with the genetic distance based clustering and PCoA. Analysis of rice accessions, both aromatic and non-aromatic, from NE Indian states revealed 2–4 clusters. Based on morphological analysis, Vairavan et al. [39] reported three clusters within a set of 400 accessions from NE India. Similar grouping was obtained by analysing 6,984 NE Indian rice accessions using SNP markers [33]. Choudhury et al. [31] reported two clusters within 24 indigenous and improved rice varieties of NE India, while Das et al. [32] found four groups among a set of 26 rice cultivars. The rice population dynamics is inextricably linked to human activities. In addition to the biological determinants, farmers’ management practices and preferences greatly influence the distribution of genetic diversity and the levels of gene flow. In the present study, it is apparent that aromatic landraces grown in Assam (*joha*), Mizoram (*tai*) and Sikkim are mostly short to medium in kernel length, bold in kernel shape and strongly aromatic. These landraces are both genetically and morphologically distinct from those of Manipur (*chakhao*) and Nagaland. Moreover, the aromatic landraces of Manipur are distinct from that of Nagaland. Combined analysis of NE Indian and global rice accessions using multiple approaches including model-based grouping, NJ tree and PCoA confirmed the assignment of the aromatic accessions from Assam, Manipur and Sikkim to the *Indica* group, while the accessions from Nagaland exhibited a close association with *Japonica*. The *japonica* cultivars have overall lower genetic diversity than *indica* [8,40,41]. In the present study, the genetic diversity within Nagaland accessions was also lower than the values recorded in other groups. The *tai* accessions of Mizoram along with few *chakhao* accessions collected from the hill districts of Manipur have been identified as admixed. Although STRUCTURE analysis clearly identified the uppermost level of structure for the populations studied here, the difficulties encountered in determining the optimal value of *K* may obscure the detection of further sub-structuring with real biological significance. From the overall results, it is proposed that the *joha*, *tai* and aromatic accessions of Sikkim are intermediate accessions between *aus* and *aromatic*. The *chakhao* accessions are aromatic *indicas*. The aromatic landraces from Nagaland can be classified as *temperate japonicas* based on their position in the NJ tree and PCoA and their lower pair-wise *F*_*ST*_ values with *temperate japonicas* in comparison to *tropical japonicas*.

In conclusion, SSR marker based molecular characterization of NE Indian aromatic and quality rice landraces revealed that large variation exists among the accessions. The population structure analysis identified three major groups within these landraces which largely correlate with the state-wise grouping as well as farmers’ classification of the landraces. Future population genetics-based studies including extensive collections of rice genetic resources from all the states of NE India will help in exploiting this rice gene pool more effectively in rice improvement programmes. In both domestic and international markets Basmati rices command premium prices, typically two to three times higher than traditional aromatic and quality rice cultivars due to consumer quality preference. Some of the traditional aromatic rice of NE India such as *chakhao* cultivars of the state of Manipur has export potentiality in South-east Asian countries where consumers prefer sticky rice. In contrast to Basmati rices, the traditional aromatic landraces can be used for a variety of cuisine starting from table rice to deserts, from offerings in various religious ceremonies and social gatherings to diet for the convalescent [32]. These landraces are also used for making popped rice. Greater appreciation of the genetic diversity preserved in the aromatic rice gene pool of NE India will facilitate proper maintenance, conservation and utilization of this valuable resource.

## Supporting Information

S1 Fig Evaluation of STRUCTURE outputs for aromatic and quality rice landraces from North-eastern India. (**a**) Mean LnP(D) over five runs for each *K* value. (**b**) Rate of change in the log probability of data between successive *K* values (Δ*K*).
(TIF)

S2 Fig Evaluation of STRUCTURE outputs for the combined dataset including aromatic and quality rice landraces from North-eastern India and global rice cultivars. (**a**) Mean LnP(D) over five runs for each *K* value. (**b**) Rate of change in the log probability of data between successive *K* values (Δ*K*).
(TIF)

S1 Table Information on the aromatic and quality rice accessions of North-eastern India used in this study. (DOC)

S2 Table Details of global rice cultivars taken from Garris et al., 2005. (DOC)

S3 Table Genotyping data of 107 aromatic and quality rice landraces of North-eastern India. (XLS)

S4 Table Population pair-wise comparisons. (DOC)
