# Correction: Genetic Diversity and Population Structure in Aromatic and Quality Rice (*Oryza sativa* L.) Landraces from North-Eastern India

**DOI:** 10.1371/journal.pone.0141405

**Published:** 2015-10-20

**Authors:** Somnath Roy, Amrita Banerjee, Bandapkuper Mawkhlieng, A. K. Misra, A. Pattanayak, G. D. Harish, S. K. Singh, S. V. Ngachan, K. C. Bansal

There are errors in the seventh sentence of the Abstract. The correct sentence is: Population structure analysis revealed that NE Indian aromatic rice can be subdivided into three genetically distinct population clusters: P1, *joha* rice accessions from Assam, *tai* rices from Mizoram and those from Sikkim; P2, aromatic rice accessions from Nagaland; and P3, *chakhao* rice germplasm from Manipur.
