# Supplementary figures and images for: Genetic Diversity and Population Structure in Aromatic and Quality Rice (Oryza sativa L.) Landraces from North-Eastern India
Source: PLoS One. 2015 Jun 12;10(6):e0129607. doi: 10.1371/journal.pone.0129607 (PMC4467088; doi:10.1371/journal.pone.0129607)

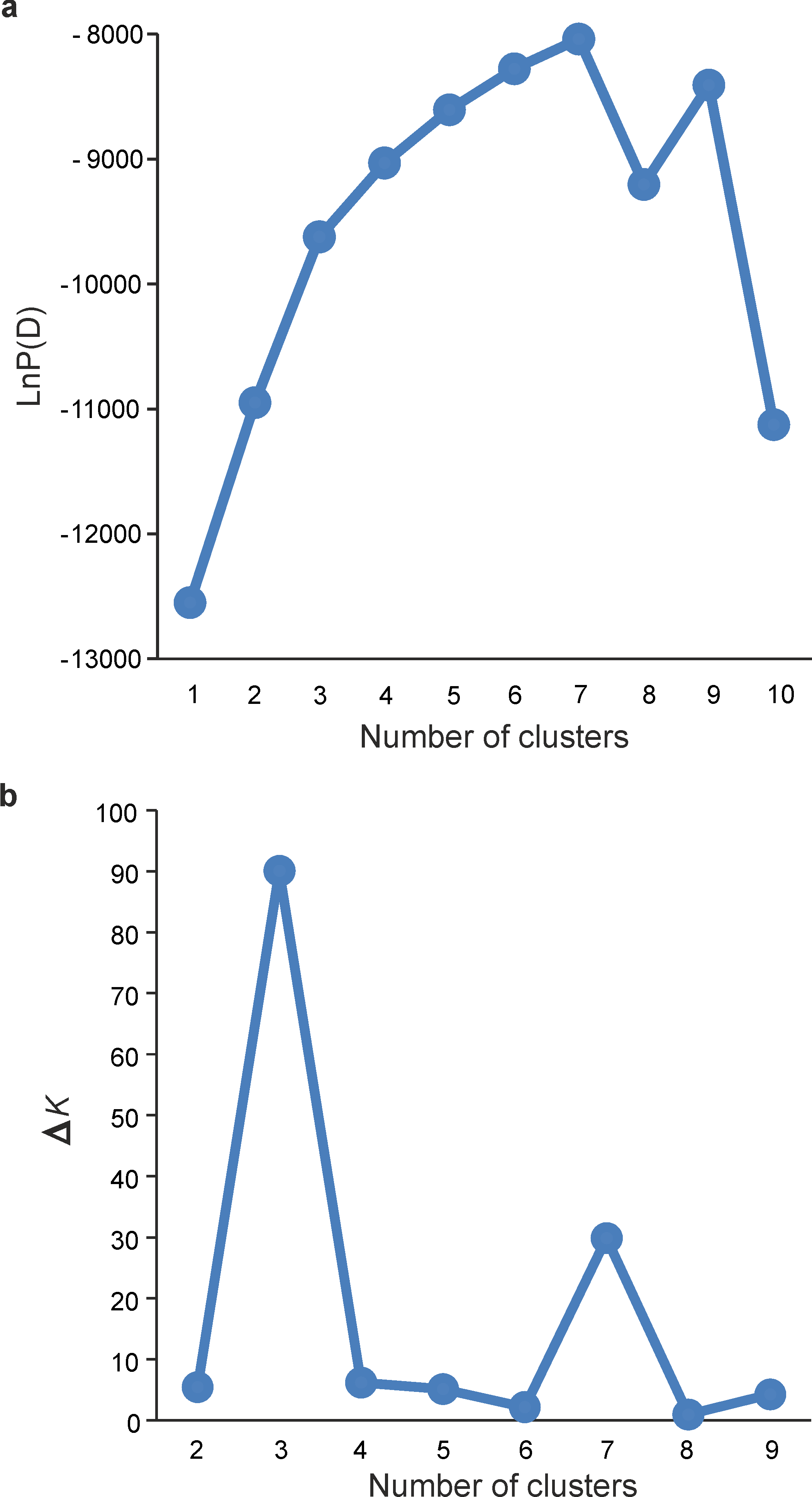

Supplement: S1 Fig — (a) Mean LnP(D) over five runs for each K value. (b) Rate of change in the log probability of data between successive K values (ΔK). (TIF) [file pone.0129607.s001.tif]

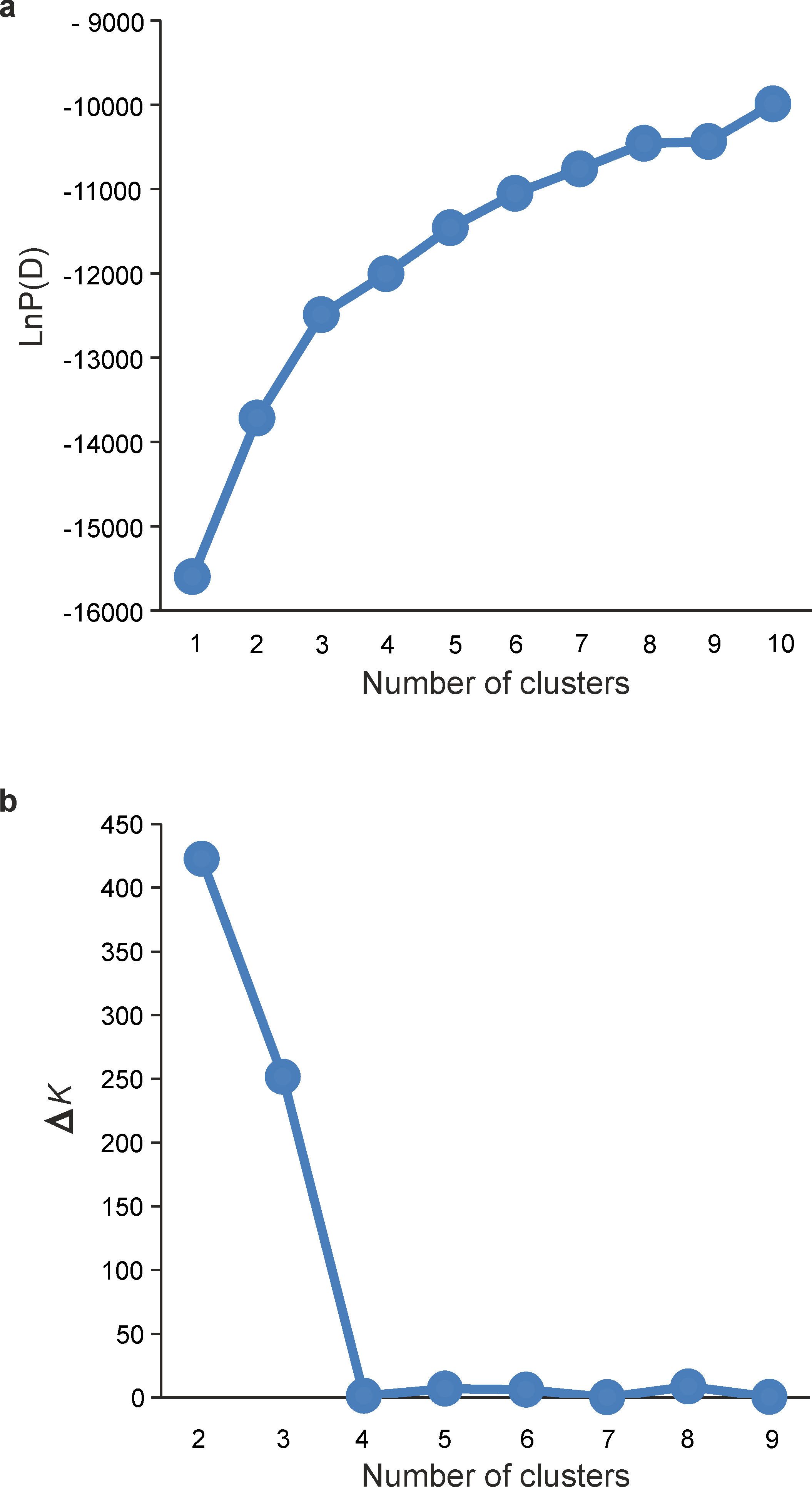

Supplement: S2 Fig — (a) Mean LnP(D) over five runs for each K value. (b) Rate of change in the log probability of data between successive K values (ΔK). (TIF) [file pone.0129607.s002.tif]
