# Supplementary material for: Genetic Diversity and Population Structure in Aromatic and Quality Rice (Oryza sativa L.) Landraces from North-Eastern India
Source: PLoS One. 2015 Jun 12;10(6):e0129607. doi: 10.1371/journal.pone.0129607 (PMC4467088; doi:10.1371/journal.pone.0129607)
Supplement: S1 Table — (DOC) [file pone.0129607.s003.doc]

**S1 Table. Information on the aromatic and quality rice accessions of North-eastern India used in this study.**

| **SN** | **Accession** | **Local Name** | **Place of collection** | **State** | **Aroma content^a^** | **Brown rice length class^b^** | **Brown rice shape class** | **Model-based group (NE Indian germplasm)** | **Model-based grouping (in relation to global accessions at *K*=2)** |
| --- | --- | --- | --- | --- | --- | --- | --- | --- | --- |
| 1 | IC450248 | Balam joha | NA | Assam | High | Medium | Bold | P1 | Admix |
| 2 | IC545129 | Joha Bora | Lakhimpur | Assam | Medium | Medium | Medium | P1 | *Indica* |
| 3 | IC545272 | Khorika joha | Dhemaji | Assam | High | Medium | Bold | P1 | *Indica* |
| 4 | IC464473 | Joha Kiik | Mikir Hills | Assam | High | Medium | Slender | P1 | *Indica* |
| 5 | IC466404 | Tikola joha | Titabar | Assam | High | Medium | Medium | P1 | *Indica* |
| 6 | IC466427 | Pokikoli joha | Mikir and NC Hills | Assam | Medium | Medium | Medium | P1 | *Indica* |
| 7 | IC466582 | Ahu joha | Kamrup | Assam | High | Medium | Bold | P1 | *Indica* |
| 8 | IC466973 | Solong joha | Mikir Hills | Assam | High | Medium | Medium | P1 | *Indica* |
| 9 | IC280987 | Cheena joha | Nalbari | Assam | High | Short | Bold | P1 | *Indica* |
| 10 | IC264481 | Nepali joha | Dhubri | Assam | Medium | Medium | Medium | P1 | Admix |
| 11 | IC264503 | Anu joha | Dhubri | Assam | High | Short | Bold | P1 | *Indica* |
| 12 | IC323566 | Abor joha | NA | Assam | Medium | Short | Bold | P1 | *Indica* |
| 13 | IC323679 | Bengali joha | NA | Assam | High | Medium | Medium | P1 | *Indica* |
| 14 | IC323682 | Boga joha | NA | Assam | Medium | Medium | Medium | P1 | *Indica* |
| 15 | IC323683 | Bhugri joha | NA | Assam | High | Medium | Medium | P1 | *Indica* |
| 16 | IC323685 | Bogi joha | NA | Assam | Medium | Medium | Medium | P1 | *Indica* |
| 17 | IC323700 | Goal Pari joha | NA | Assam | Medium | Medium | Medium | P1 | *Indica* |
| 18 | IC323712 | Kala joha | NA | Assam | High | Medium | Medium | P1 | *Indica* |
| 19 | IC323713 | Joha Bora | NA | Assam | High | Short | Medium | P1 | *Indica* |
| 20 | IC323725 | Kala joha | NA | Assam | High | Short | Bold | P1 | *Indica* |
| 21 | IC323730 | Koli joha | NA | Assam | High | Short | Bold | P1 | *Indica* |
| 22 | IC323732 | Kon joha | NA | Assam | High | Short | Bold | P1 | *Indica* |
| 23 | IC323735 | Kunkuni joha | NA | Assam | High | Short | Medium | P1 | *Indica* |
| 24 | IC323736 | Kopousali joha | NA | Assam | High | Medium | Medium | P1 | *Indica* |
| 25 | IC323737 | Kon Bogi joha | NA | Assam | Medium | Medium | Medium | P1 | *Indica* |
| 26 | IC323738 | Khorika joha | NA | Assam | Medium | Medium | Medium | P1 | *Indica* |
| 27 | IC323739 | Kamini joha | NA | Assam | High | Short | Medium | P1 | *Indica* |
| 28 | IC323741 | Khudi joha | NA | Assam | High | Short | Medium | P1 | *Indica* |
| 29 | IC323767 | Nepali joha | NA | Assam | Medium | Extra long | Slender | P1 | *Indica* |
| 30 | IC352785 | Kajala joha | NA | Assam | High | Short | Bold | P1 | *Indica* |
| 31 | IC352812 | Chand joha | Bongaigaon | Assam | High | Medium | Medium | P1 | *Indica* |
| 32 | IC380576 | Hogga joha | Golaghat | Assam | Medium | Medium | Medium | P1 | *Indica* |
| 33 | IC380624 | Sana joha | Bongaigaon | Assam | High | Short | Bold | P1 | *Indica* |
| 34 | IC380667 | Keteki joha | Karimganj | Assam | Medium | Long | Medium | P1 | *Indica* |
| 35 | IC394328 | Gandheli joha | Dhemaji | Assam | High | Short | Bold | P1 | *Indica* |
| 36 | IC394495 | Basmati joha | Dibrugarh | Assam | High | Long | Slender | P1 | *Indica* |
| 37 | IC394502 | Maniki madhuri joha | Dibrugarh | Assam | High | Short | Medium | P1 | *Indica* |
| 38 | IC394698 | Chahab joha | Sonitpur | Assam | Medium | Long | Medium | P1 | *Indica* |
| 39 | IC394700 | Charkari joha | Sonitpur | Assam | Medium | Medium | Medium | P1 | *Indica* |
| 40 | IC423084 | Krishna joha | Sonitpur | Assam | High | Long | Slender | P1 | *Indica* |
| 41 | IC423091 | Ronga joha | Sonitpur | Assam | Medium | Medium | Medium | P1 | *Indica* |
| 42 | IC423106 | Pokarpura joha | Sonitpur | Assam | High | Medium | Slender | P1 | *Indica* |
| 43 | IC538301 | Punjab joha | Unknown | Assam | High | Medium | Medium | P1 | *Indica* |
| 44 | IC319352 | Kola joha | Sonitpur | Assam | High | Medium | Medium | P1 | *Indica* |
| 45 | IC394784 | Bormoni joha | Morigaon | Assam | Medium | Long | Slender | P1 | *Indica* |
| 46 | IC394796 | Kunkuni joha | Morigaon | Assam | High | Short | Bold | P1 | Admix |
| 47 | IC352805 | Kothari joha | Bongaigaon | Assam | Medium | Medium | Medium | P1 | *Indica* |
| 48 | IC324099 | Khorika joha | Darrang | Assam | High | Medium | Medium | P1 | *Indica* |
| 49 | IC332887 | Ronga joha | Dhemaji | Assam | High | Long | Medium | P1 | *Indica* |
| 50 | IC332955 | Baboli joha | Dhemaji | Assam | High | Medium | Medium | P1 | *Indica* |
| 51 | IC332973 | Gandheli joha | Dhemaji | Assam | High | Medium | Medium | P1 | *Indica* |
| 52 | IC333026 | Mem joha | Sonitpur | Assam | High | Long | Medium | P1 | *Indica* |
| 53 | IC333032 | Joha Kiying | Sonitpur | Assam | High | Short | Medium | P1 | *Indica* |
| 54 | IC324094 | Bhog joha | Darrang | Assam | Medium | Long | Medium | P1 | *Indica* |
| 55 | IC554799 | Krishna bhog-1 | East Sikkim | Sikkim | Medium | Long | Medium | P1 | *Indica* |
| 56 | IC554802 | Kalanunia | East Sikkim | Sikkim | High | Medium | Slender | P1 | *Indica* |
| 57 | IC554816 | Baghe tulashi | East Sikkim | Sikkim | Medium | Short | Bold | P1 | *Indica* |
| 58 | IC554841 | Krishna bhog-2 | East Sikkim | Sikkim | Medium | Long | Slender | P1 | *Indica* |
| 59 | IC441468 | Brimphul | Mangan | Sikkim | High | Medium | Bold | P1 | *Indica* |
| 83 | IC265353 | Tai Klawnglawh | Kolasib | Mizoram | Medium | Long | Medium | P1 | Admix |
| 84 | IC265398 | Vaiphei tai | Kolasib | Mizoram | High | Medium | Medium | P1 | Admix |
| 85 | IC265400 | Tai Farete | Kolasib | Mizoram | Medium | Long | Medium | P1 | Admix |
| 86 | IC265352 | Buh tai-1 | Kolasib | Mizoram | High | Extra long | Medium | P1 | Admix |
| 87 | IC265411 | Kowonglawng tai | Kolasib | Mizoram | High | Long | Bold | P1 | Admix |
| 88 | IC265458 | Tai-1 | Kolasib | Mizoram | Medium | Medium | Bold | P1 | Admix |
| 89 | IC265490 | Tai Kawlang | Kolasib | Mizoram | Medium | Long | Bold | P1 | Admix |
| 90 | IC297694 | Tai-2 | Chhamphai | Mizoram | High | Short | Bold | P1 | *Indica* |
| 91 | IC297715 | Tailte | Lunglei | Mizoram | Medium | Medium | Medium | P1 | Admix |
| 68 | MRS-51 | Amo | Lohit | Arunachal Pradesh | Medium | Medium | Medium | P1 | Admix |
| 70 | MRS-58 | Khaw | Lohit | Arunachal Pradesh | Medium | Medium | Bold | P2 | *Japonica* |
| 60 | IC0596612 | Thekieniera | Kohima | Nagaland | Medium | Extra long | Medium | P2 | *Japonica* |
| 61 | B.P.B/I.W/S.N-15 | Lharuno | Kohima | Nagaland | Medium | Medium | Bold | P2 | *Japonica* |
| 62 | IC0596622 | Omang tsuk | Mokokchung | Nagaland | Medium | Medium | Bold | P2 | *Japonica* |
| 63 | B.P.B/K.E/J.K.S-32 | Ingra maro | Mokokchung | Nagaland | Medium | Extra long | Medium | P2 | *Japonica* |
| 64 | B.P.B/K.E/J.K.S-33 | Longratsuk | Mokokchung | Nagaland | Low | Medium | Bold | P2 | *Japonica* |
| 65 | B.P.B/K.E/J.K.S-48 | Sheshanga | Wokha | Nagaland | Medium | Long | Bold | P2 | *Japonica* |
| 66 | IC0596634 | Shiku tsuk | Mokokchung | Nagaland | Medium | Extra long | Bold | P2 | *Japonica* |
| 67 | B.P.B/K.E/J.K.S-47 | Jaha ekyu | Wokha | Nagaland | Medium | Long | Medium | P2 | *Japonica* |
| 69 | B.P.B/K.E/J.K.S-53 | Shesharga nuko | Wokha | Nagaland | Medium | Extra long | Medium | P2 | *Japonica* |
| 71 | B.P.B/K.E/J.K.S-59 | Otruk enyiko | Wokha | Nagaland | Low | Long | Bold | P2 | *Japonica* |
| 72 | IC0596639 | Chopru ekyu | Wokha | Nagaland | Medium | Extra long | Medium | P2 | *Japonica* |
| 73 | B.P.B/K.E/J.K.S-61 | Rekhathung shopruo | Wokha | Nagaland | Medium | Long | Bold | P2 | *Japonica* |
| 74 | IC0596646 | Sonporo ekyov | Wokha | Nagaland | Medium | Extra long | Medium | P2 | *Japonica* |
| 75 | IC0596648 | Mecheying | Wokha | Nagaland | Low | Extra long | Bold | P2 | *Japonica* |
| 76 | IC0596649 | Eramoren tsuknyiku | Wokha | Nagaland | Medium | Long | Bold | P2 | *Japonica* |
| 77 | B.P.B/K.E/J.K.S-73 | Vadvu tsuk | Wokha | Nagaland | Medium | Long | Bold | P2 | *Japonica* |
| 78 | B.P.B/K.E/J.K.S-74 | Yepvu tsuk | Wokha | Nagaland | Medium | Long | Bold | P2 | *Japonica* |
| 79 | IC0596650 | Jhuv ekyov | Wokha | Nagaland | Low | Extra long | Medium | P2 | *Japonica* |
| 80 | IC0596651 | Tsokunkvu | Wokha | Nagaland | Low | Extra long | Medium | P2 | *Japonica* |
| 81 | B.P.B/K.E/J.K.S-77 | Wokhae kendemo | Wokha | Nagaland | Low | Extra long | Bold | P2 | *Japonica* |
| 82 | B.P.B/ K.R -102 | Changman san | Dimapur | Nagaland | Medium | Extra long | Medium | P2 | *Japonica* |
| 92 | IC0596559 | Chakhao poireiton | Imphal West | Manipur | High | Long | Medium | P3 | *Indica* |
| 93 | IC0596560 | Chakhao angouba | Imphal West | Manipur | High | Short | Bold | P3 | *Indica* |
| 94 | SRMN3 | Chakhao amubi | Imphal West | Manipur | High | Medium | Medium | P3 | *Indica* |
| 95 | IC0596561 | Langphou angouba | Imphal West | Manipur | Low | Medium | Medium | P3 | *Indica* |
| 96 | IC0596565 | Chakhao poireiton | Thoubal | Manipur | High | Medium | Medium | P3 | *Indica* |
| 97 | IC0596566 | Chakhao amubi | Thoubal | Manipur | High | Medium | Medium | P3 | *Indica* |
| 98 | IC0596571 | Chakhao angouba | Thoubal | Manipur | Medium | Long | Medium | P3 | *Indica* |
| 99 | IC0596575 | Kathaibuw | Chandel | Manipur | Low | Long | Medium | P3 | Admix |
| 100 | IC0596577 | Buhman | Churachandpur | Manipur | High | Long | Medium | P3 | *Indica* |
| 101 | IC0596578 | Buhman | Churachandpur | Manipur | Medium | Long | Slender | P3 | *Indica* |
| 102 | IC0596583 | Napnang hangmei | Tamenglong | Manipur | Medium | Long | Medium | P3 | Admix |
| 103 | IC0596585 | Chakhao phou | Tamenglong | Manipur | High | Long | Medium | P3 | Admix |
| 104 | IC0596588 | Chakhao | Bishnupur | Manipur | High | Medium | Slender | P3 | Indica |
| 105 | IC0596591 | Makrei | Ukhrul | Manipur | Medium | Medium | Medium | P3 | Admix |
| 106 | IC0596593 | Makrei | Ukhrul | Manipur | Medium | Medium | Medium | P3 | Indica |
| 107 | SRMN41 | Manui maa | Ukhrul | Manipur | Medium | Long | Medium | P3 | Admix |

Notes: IC, Indigenous collection; NA, not available

^a^Grain aroma content detected according to Nagaraju et al. [1]

^b,c^Determined following International Rice Research Institute (IRRI) Standard evaluation system for rice [2]

**References:**

1. Nagaraju M, Mohanty KK, Chaudhary D, Gangadharan CA (1991) Simple technique to detect scent in rice. Oryza 28:109-110
2. IRRI (1996) Standard Evaluation System for Rice. Los Banos, IRRI
