# Supplementary material for: Genetic Diversity and Population Structure in Aromatic and Quality Rice (Oryza sativa L.) Landraces from North-Eastern India
Source: PLoS One. 2015 Jun 12;10(6):e0129607. doi: 10.1371/journal.pone.0129607 (PMC4467088; doi:10.1371/journal.pone.0129607)
Supplement: S2 Table — (DOC) [file pone.0129607.s004.doc]

**S2 Table. Details of global rice cultivars taken from Garris et al. (2005).**

| **SN** | **Accession name** | **IRGC#*** | **Model-based group** | **Country of origin** |
| --- | --- | --- | --- | --- |
| 1 | ARC 10177 | IRGC12386 | Aus | India |
| 2 | ARC 10352 | IRGC12440 | Aus | India |
| 3 | ARC 13829 | IRGC42469 | Aromatic | India(N. Kakhimpur) |
| 4 | ARC 7229 | IRGC12331 | Admixture (AUS,TRJ,TEJ) | India |
| 5 | Baber | IRGC33984 | Temperate japonica | India |
| 6 | Badkalamkati | IRGC45011 | Indica | India |
| 7 | Basmati | PI385418 | Aromatic | Pakistan |
| 8 | Basmati 1 | IRGC27798 | Aus | Pakistan |
| 9 | Basmati 217 | IRGC53637 | Aromatic | India(Punjab) |
| 10 | Bhadoia 233 | IRGC6541 | Aus | Bangladesh |
| 11 | BJ1 | IRGC45195 | Aus | India |
| 12 | Black Gora | IRGC40275 | Aus | India |
| 13 | Chhote Dhan | IRGC58930 | Indica | Nepal |
| 14 | Chitraj(DA23) | IRGC6208 | Indica | Bangladesh |
| 15 | CO18 | IRGC6331 | Indica | India |
| 16 | DA13 | IRGC5857 | Aromatic | Bangladesh |
| 17 | DA16 | IRGC6245 | Admixture (AUS,IND) | Bangladesh |
| 18 | Darmali | IRGC27630 | Temperate japonica | Nepal |
| 19 | Davao | IRGC8244 | Tropical japonica | Phillipines |
| 20 | Dhala Shaitta | PI180060 | Aus | Bangladesh |
| 21 | Dhola Aman | IRGC8341 | Indica | Bangladesh |
| 22 | Dholi Boro | IRGC27513 | Tropical japonica | Bangladesh |
| 23 | Dular | IRGC32561 | Admixture (TRJ,TEJ) | India |
| 24 | DV85 | IRGC8839 | Aus | Bangladesh |
| 25 | DZ78 | IRGC8555 | Aus | Bangladesh |
| 26 | FR13 A | IRGC6144 | Aus | India |
| 27 | IR36 | IRGC30416 | Indica | Phillipines |
| 28 | IR8 | PI312627 | Indica | Phillipines |
| 29 | Jaya | PI430261 | Indica | India |
| 30 | JC1 | IRGC9091 | Aromatic | India |
| 31 | JC101 | IRGC9060 | Aromatic | India |
| 32 | JC111 | IRGC9062 | Aromatic | India |
| 33 | JC148 | IRGC9069 | Aus | India |
| 34 | JC149 | IRGC9070 | Aromatic | India |
| 35 | JC157 | IRGC9179 | Aromatic | India |
| 36 | JC73-4 | IRGC9115 | Aromatic | India |
| 37 | Jhona 349 | IRGC6307 | Aus | India |
| 38 | Jumali | IRGC9542 | Admixture (ARO,TEJ) | Nepal |
| 39 | Kalamkati | IRGC45975 | Aus | India |
| 40 | Kasalath | HO1195 | Aus | India |
| 41 | Kinastano | IRGC3782 | Tropical japonica | Philippines |
| 42 | Lal Aman | IRGC46202 | Indica | India |
| 43 | Miriti | IRGC25901 | Tropical japonica | Bangladesh |
| 44 | MTU9 | IRGC7919 | Indica | India |
| 45 | Mudgo | IRGC6663 | Indica | India |
| 46 | N12 | IRGC6298 | Aromatic | India |
| 47 | N22 | IRGC6264 | Tropical japonica | India |
| 48 | NHTA 10 | IRGC191 | Admixture (TRJ,TEJ) | India |
| 49 | NPE 253 | IRGC38690 | Temperate japonica | Pakistan |
| 50 | NPE 417 | IRGC38692 | Temperate japonica | Pakistan |
| 51 | NPE 826 | IRGC38694 | Temperate japonica | Pakistan |
| 52 | NPE 835 | IRGC38696 | Temperate japonica | Pakistan |
| 53 | NPE 844 | IRGC38698 | Tropical japonica | Pakistan |
| 54 | Pankhari 203 | IRGC5999 | Aromatic | India |
| 55 | Patnai 23 | IRGC46531 | Indica | India |
| 56 | Peh-Ni-Nuo | IRGC8266 | Admixture (TRJ,TEJ) | China |
| 57 | Phudugey | IRGC32399 | Aus | Bhutan |
| 58 | PTB9 | IRGC6274 | Indica | India |
| 59 | Rikuto Kemochi | IRGC2719 | Admixture (TRJ,IND) | Japan |
| 60 | Shan Kiu Ju | IRGC1154 | Admixture (TRJ,TEJ) | China |
| 61 | T1 | IRGC6294 | Aus | India |
| 62 | T26 | IRGC46768 | Admixture (AUS,ARO,IND) | India |
| 63 | Ta Hung Ku | IRGC1107 | Temperate japonica | China |
| 64 | Ta Mao Tsao | IRGC8194 | Temperate japonica | China |
| 65 | TKM6 | IRGC237 | Indica | India |
| 66 | WC6 | CIor5309 | Temperate japonica | Jiangsu, China |
| 67 | Yelaik Meedon | IRGC33888 | Temperate japonica | Burma |

Note: *International Rice Germplasm Collection at IRRI in the Philippines.
