# Supplementary material for: Genetic Diversity and Population Structure in Aromatic and Quality Rice (Oryza sativa L.) Landraces from North-Eastern India
Source: PLoS One. 2015 Jun 12;10(6):e0129607. doi: 10.1371/journal.pone.0129607 (PMC4467088; doi:10.1371/journal.pone.0129607)
Supplement: S4 Table — (DOC) [file pone.0129607.s006.doc]

**S4 Table. Population pair-wise comparisons.**

| **Groups** | **P1** | **P2** | **P3** | ***admixed*** | ***aromatic*** | ***aus*** | ***indica*** | ***TEJ*** | ***TRJ*** |
| --- | --- | --- | --- | --- | --- | --- | --- | --- | --- |
| P1 |  | 0.351^***^ | 0.223^***^ | - | - | - | - | - | - |
| P2 | 0.366^***^ |  | 0.453^***^ | - | - | - | - | - | - |
| P3 | 0.217^***^ | 0.441^***^ |  | - | - | - | - | - | - |
| *admix* | 0.148^***^ | 0.355^***^ | 0.211^***^ |  | - | - | - | - | - |
| *aromatic* | 0.186^***^ | 0.412^***^ | 0.271^***^ | 0.186^***^ |  | - | - | - | - |
| *aus* | 0.150^***^ | 0.506^***^ | 0.219^***^ | 0.208^***^ | 0.283^***^ |  | - | - | - |
| *indica* | 0.252^***^ | 0.542^***^ | 0.227^***^ | 0.262^***^ | 0.378^***^ | 0.201^***^ |  | - | - |
| *TEJ* | 0.300^***^ | 0.382^***^ | 0.356^***^ | 0.150^***^ | 0.314^***^ | 0.393^***^ | 0.443^***^ |  | - |
| *TRJ* | 0.301^***^ | 0.452^***^ | 0.344^***^ | 0.194^***^ | 0.327^***^ | 0.374^***^ | 0.405^***^ | 0.282^***^ |  |

Notes: Upper diagonal: *FST* values among three groups in 107 accessions of NE India. Lowe diagonal: *FST* values among 9 groups of NE Indian and global rice accessions.

*TEJ*, *temperate japonica*; *TRJ*, *tropical japonica.*

^***^ *P*<0.001
